# Effect of wood biomass components on self-heating

**DOI:** 10.1186/s40643-021-00373-7

**Published:** 2021-02-27

**Authors:** Nozomi Miyawaki, Takashi Fukushima, Takafumi Mizuno, Miyao Inoue, Kenji Takisawa

**Affiliations:** 1 Graduate School of Bioresources, Mie University, 1577 Kurimamachiyacho, Tsu, Mie 514-8507 Japan; 2 Faculty of Home Economics, Tokyo Kasei University, 1-18-1 Kaga, Itabashi, Tokyo 173-8602 Japan

**Keywords:** Wood biomass, Self-heating, Inorganic matter, Spontaneous ignition

## Abstract

Biomass may ignite due to biological oxidation and chemical oxidation. If this phenomenon (spontaneous ignition) is controlled, it would be possible to produce biochar at a lower cost without the need for an external heat resource. We investigated if self-heating could be controlled by using sawdust and bark chips. When sawdust and bark chips were used under controlled conditions, the bark chips temperature increased to the torrefaction temperature. The ash content of bark chips was ~ 2%d.b. higher than that of sawdust; consequently, the inorganic substances contained in the bark chips might affect the self-heating. Self-heating was suppressed when inorganic substances were removed by washing with water. Therefore, the inorganic substances in the biomass might have affected self-heating. The inorganic element contents of the bark chips were measured by inductively coupled plasma optical emission spectrometry before and after washing. The potassium content of the bark chips was reduced remarkably by washing, and there was a possible influence of potassium on self-heating. Finally, the effect of moisture content on self-heating was investigated to obtain stable reactivity. Thus, at a moisture content of 40%w.b., a steady self-heating behavior may be realized.
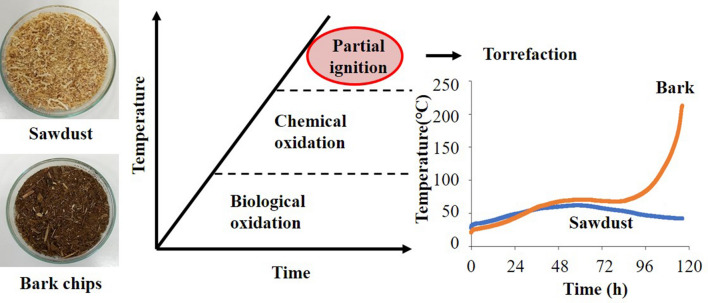

## Introduction

It is essential to understand how to handle, store, and process coal and biomass resources in processing plants. Accumulated coal, refuse-derived fuel, and biomass cause self-heating and spontaneous ignition (Wang et al. 2003a; Fu et al. 2005; Torrent et al. 2015). According to García-Torrent et al. (2012), self-ignition might account for 12% of fires in underground mines and 6% of fires in the agri-food industry. These reactions can lead to fire hazards and the release of toxic gases, which are dangerous to human health (Wang et al. 2003b; Itoh et al. 2018). Therefore, the retardation of spontaneous combustion is highly desirable. Self-heating ignition is caused by spontaneous exothermic reactions in an oxidative atmosphere at low ambient temperatures (Bowes 1984). In particular, chemical oxidation at low temperatures is the main factor that results in the spontaneous ignition of coal and relies on many factors, such as temperature, particle size, surface area, coal-pore structure, moisture content, coal rank, and the composition of ambient air (Nugroho et al. 2000). In addition, raw materials including refuse-derived fuel and biomass decompose easily. Li et al. (2006) reported that biological oxidation played an important role in inducing the spontaneous ignition of accumulated wood chips. Thus, the factors that cause self-heating ignition such as chemical oxidation and microbial activity are necessary to be controlled accurately because the resulting fires cause damage to the energy industry and health. In fact, the objectives of most researches related to self-heating ignition are to prevent the spontaneous ignition of piled materials (Ramírez et al. 2010; Wu et al. 2015; Ashman et al. 2018).

Itoh et al. (2019) reported a new biochar production method utilizing spontaneous self-heating induced by low-temperature oxidation of biomass. In this method, manure temperature control, by self-heating under elevated pressure, achieved the torrefaction temperature, which resulted in biochar production with a reduced environmental impact. Torrefaction is generally defined as a mild pyrolysis method that operates in the range of 200–300 °C (Van der Stelt et al. 2011). Torrefaction has various benefits, including higher energy density, lower moisture content, higher hydrophobicity, enhanced grindability, and more uniform properties of biomass (Chen et al. 2015). The biochar produced by torrefaction had characteristics intermediate from the raw material and coal. Biochar can be used as an adsorbent, catalyst support (Shen 2015), fertilizer, soil conditioner (Keskinen et al. 2019), carbon sequestration agents (Abdel-Fattah et al. 2015), and energy.

This study investigated biochar production using the self-heating of wood biomass under atmospheric conditions. This system might enable the production of biochar at a lower cost without the need for an external heat resource. Sawdust and bark chips were used as raw materials in this study. Both materials are representative of wood biomass. First, the self-heating conditions were compared between the sawdust and bark chips of cedar to test the suitability of the wood species for self-heating. Subsequently, the effect of inorganic matter on self-heating was investigated using bark chips.

## Materials and methods

### Materials

**Fig. 1:**
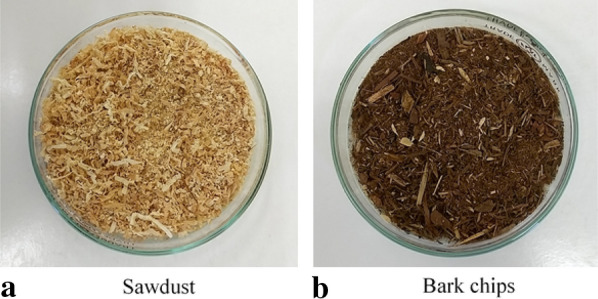
Cedar sawdust and bark chips

Cedar sawdust and bark chips (Mie, Japan) were collected as feedstock (Fig. 1). The bark chips were ground using a grinder (YKB, AS ONE Corp., Japan). Each material was dried at 105 °C for 24 h and stored until the experiment. There was almost no difference in the higher heating value, but the ash content was greater in the bark chips (Table 1). Previous studies have shown that the bark ash content tends to be greater than for sawdust regardless of the tree species, in addition to the higher heating value of sawdust being 19.6–20.5 MJ/kg while the higher heating value of bark was 17.8–20.6 MJ/kg (Kingiri et al. 1999). Similar results were obtained in the present study.

### Experimental system

**Fig. 2:**
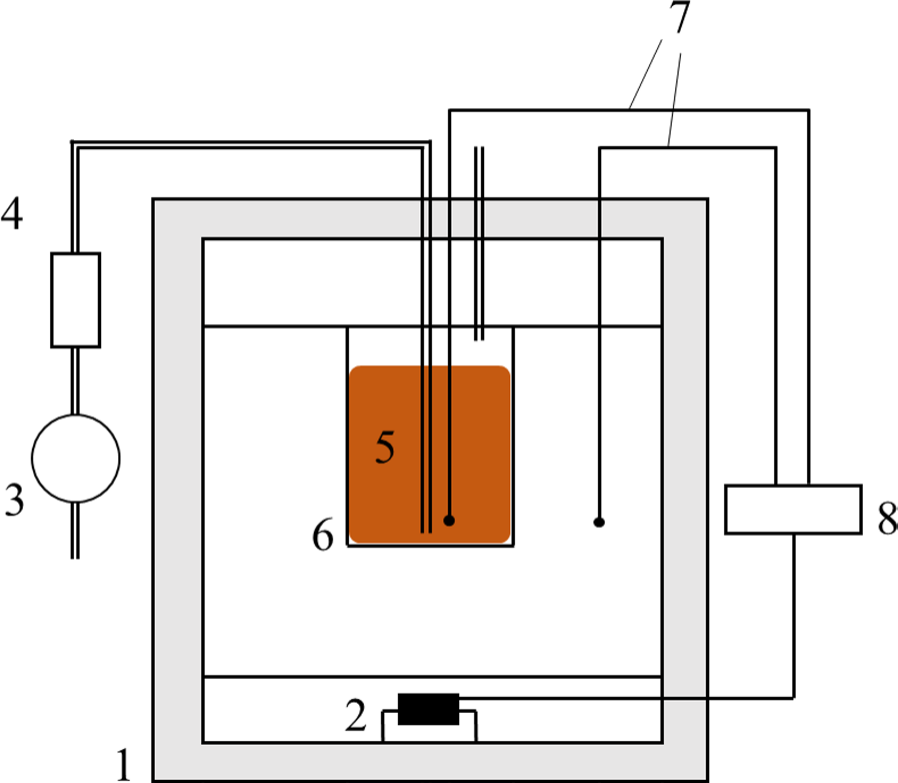
Schematic illustration of the experimental system. (1) Thermostat, (2) heater, (3) air compressor, (4) air flow meter, (5) material, (6) reactor, (7) thermocouple, (8) data logger

A schematic of the experimental apparatus is shown in Fig. 2. A thermostat (DX302, Yamato Scientific co., ltd., Japan), pump (MV-6005P, E.M.P-Japan Ltd., Japan), flow meter (RK1200, KOFLOC, Japan), and thermo recorder (TR-75wf, T&D Corp., Japan) were used for the experiments. During the experiment, the temperature in the thermostat and the sample temperature in the reactor were measured using a K-type thermocouple and recorded on a thermorecorder. The thermostat temperature was set to follow the sample temperature in the reactor within 1 °C.

### Self-heating experiments

The feasibility of successive reactions of biological oxidation, chemical oxidation, and spontaneous ignition was investigated to produce biochar using cedar sawdust. First, a self-heating experiment was carried out under the optimum composting aeration conditions to confirm microbial heat generation. Hong et al. (1983) reported an optimum range of 0.87–1.07 L/min kg-VM by composting between aeration levels of 0.04–3.0 L/min kg-VM. Therefore, 50 g of the sample adjusted to a moisture content of 40%w.b. was placed in the reactor, and the air was aerated at 1.0 L/min kg-VM. Next, a spontaneous ignition experiment on sawdust based on previous research (Li et al. 2006) was conducted. In this experiments, 50 g of cedar sawdust adjusted to a moisture content of 40%w.b. was placed in a reactor and heated at an aeration rate of 6.0 L/min kg-VM at 170, 180, and 190 °C, respectively. Finally, it is necessary to examine how the temperature rises due to the chemical oxidative reaction after biological oxidation. This is to explore the feasibility of the successive reactions of biological oxidation, chemical oxidation, and spontaneous ignition. Therefore, 50 g of cedar sawdust, adjusted to a moisture content of 40%w.b., was self-heated from 70 °C—the maximum composting temperature—at the aeration rates of 0.1, 0.2, 0.6, and 1.0 L/min kg-VM. This was to search for the optimum aeration rate for chemical oxidation.

Sawdust and bark chips were used as raw materials to produce biochar by self-heating. Each 50 g of the samples, adjusted to the moisture content 40%w.b., was set in the reactor, and self-heated at the aeration rate of 0.6 L/min kg-VM. In addition, the bark chips were washed with water to determine the effect of inorganic matter on self-heating by removing inorganic matter without changing the structure of the biomass. The procedure of Cen et al. (2016) was referred to for washing the bark chips; 10 g/L of bark chips were placed in deionized water, washed with a magnetic stirrer at 60 °C for 6 h, followed by drying at 105 °C for 24 h. Washed bark chips were adjusted to moisture content of 40%w.b. and self-heated at the aeration rate of 0.6 L/min kg-VM.

Stable reproducibility is essential for establishing the process. It is necessary to confirm the self-heating reproducibility because the heat generation associated with chemical oxidation is small at low temperatures. In particular, the reactivity depends on the moisture content. Thus, the self-heating experiment was conducted using 50 g of bark chips with a moisture content of 10% and 40%w.b. Each experiment was carried out at a rate of 0.6 L/min kg-VM.

### Analysis

Moisture and ash contents were determined by drying the samples at 105 °C for 24 h, followed by incineration at 600 °C for 3 h. The higher heating values were measured using an adiabatic calorimeter (OSK150, Ogawa Sampling Corp., Japan). Inductively coupled plasma optical emission spectrometry (Agilent 5110 ICP-OES, Agilent Technologies, Inc., USA) was used to analyze the composition of the inorganic substances in the samples. The samples were heated and dissolved in nitric acid and hydrogen peroxide as a pretreatment for analysis. The pretreated solutions were diluted and analyzed, and calcium, potassium, magnesium, sodium, and phosphorus standard solutions were used for inorganic analysis.

## Results and discussion

### Self-heating experiments using sawdust

**Fig. 3:**
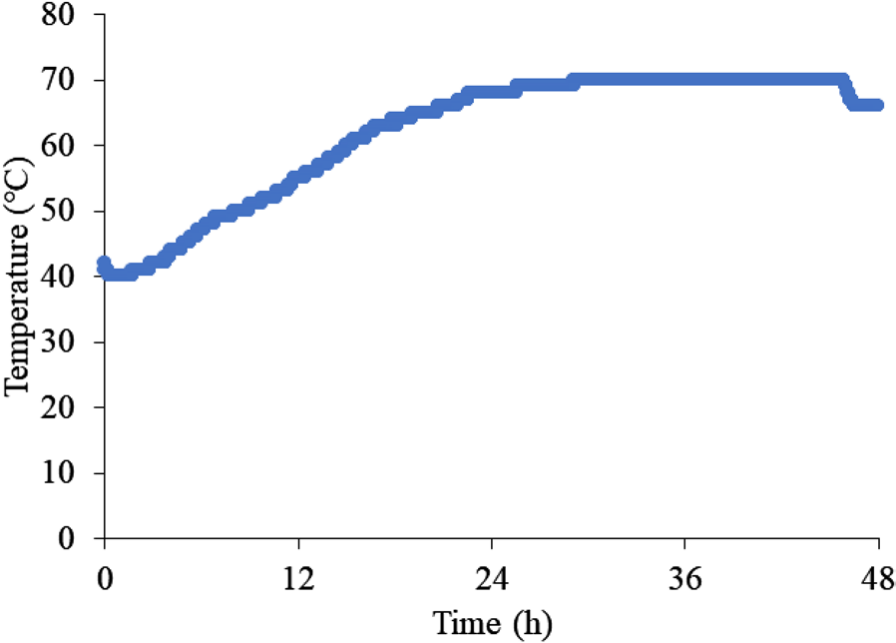
Composting of cedar sawdust

**Fig. 4:**
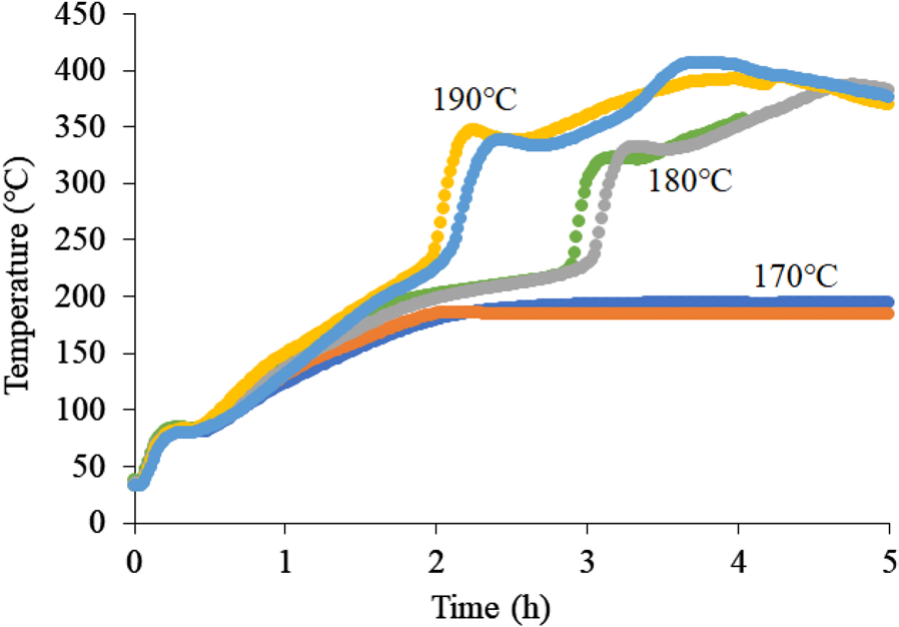
Ignition of cedar sawdust at each temperature. Each experiment was performed in duplicate

**Fig. 5:**
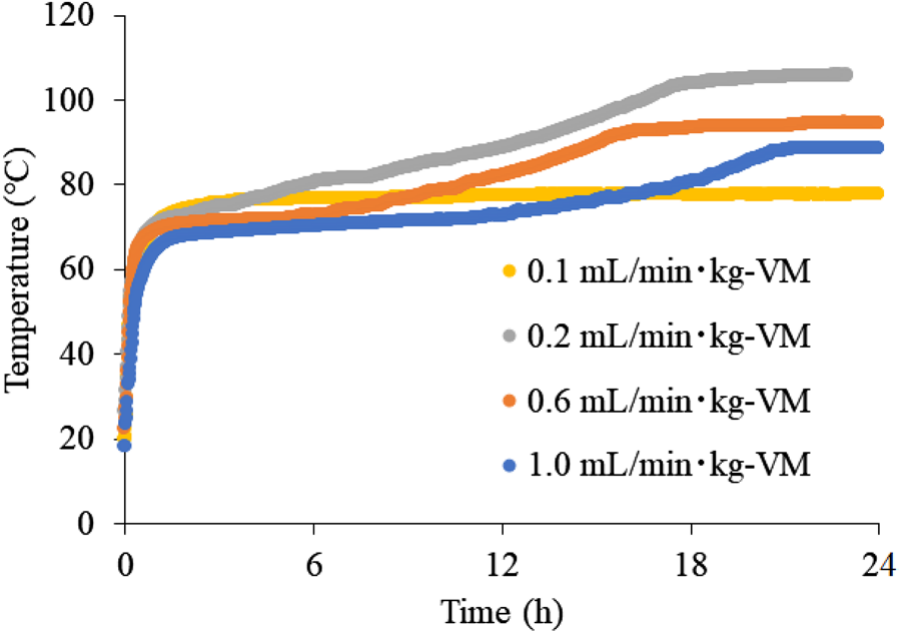
Self-heating of cedar sawdust at each aeration rate

Figure 3 shows the results of the biological oxidation experiments using cedar sawdust. The sawdust temperature reached 70 °C at ~ 30 h. From this heat generation behavior, it was confirmed that composting by microorganisms occurred successfully. The spontaneous ignition temperature was also investigated at various constant temperatures. Figure 4 shows the results of spontaneous ignition temperature experiments. No temperature rise was observed at 170 °C, but a rapid temperature rise was observed above 180 °C. This result suggests that more than 180 °C may be necessary to spontaneously ignite sawdust. Finally, the oxidation experiments were carried out from 70 °C by varying the aeration rate between 0.1 and 1.0 L/min kg-VM to increase from the composting temperature to spontaneous ignition temperature by the chemical oxidation of the sawdust. As a result, when the aeration rate was 0.2 L/min kg-VM, the highest temperature rise was observed, where the maximum temperature reached ~ 100 °C (Fig. 5). This result is thought to be due to the lack of oxygen supply at the aeration rate of 0.1 L/min kg-VM and the consumption of heat by the excess air supply at the aeration rate of more than 0.6 L/min kg-VM. Regardless, the sawdust temperature could not reach a spontaneous ignition temperature of 180 °C.

### Difference between sawdust and bark chips

**Fig. 6:**
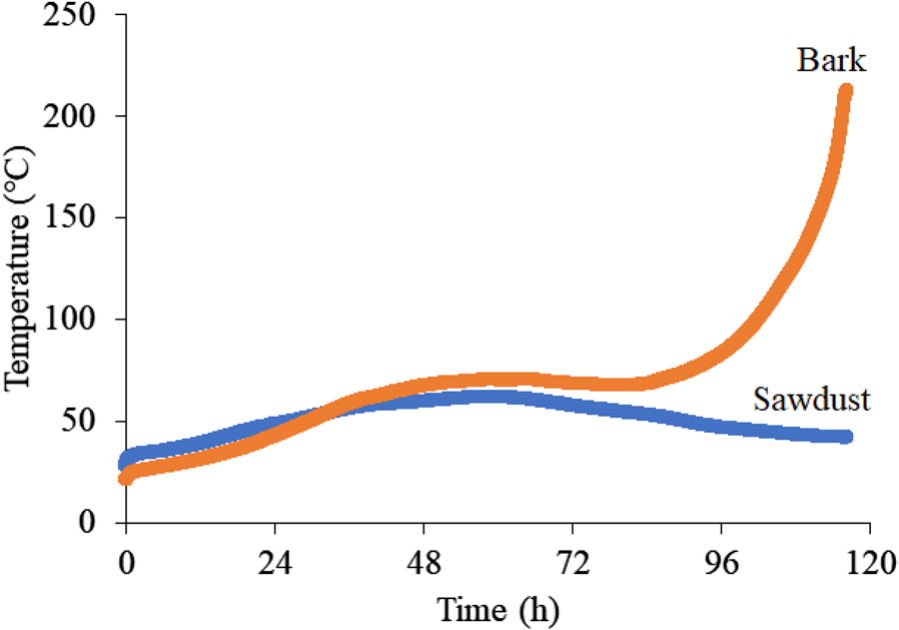
Self-heating of sawdust and bark chips

**Fig. 7:**
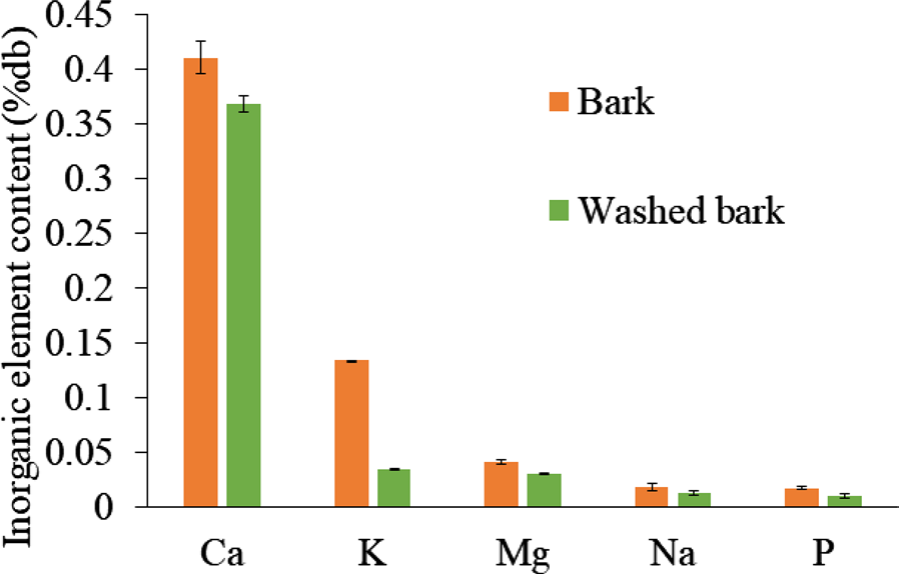
Inorganic element content in raw and washed bark chips

**Fig. 8:**
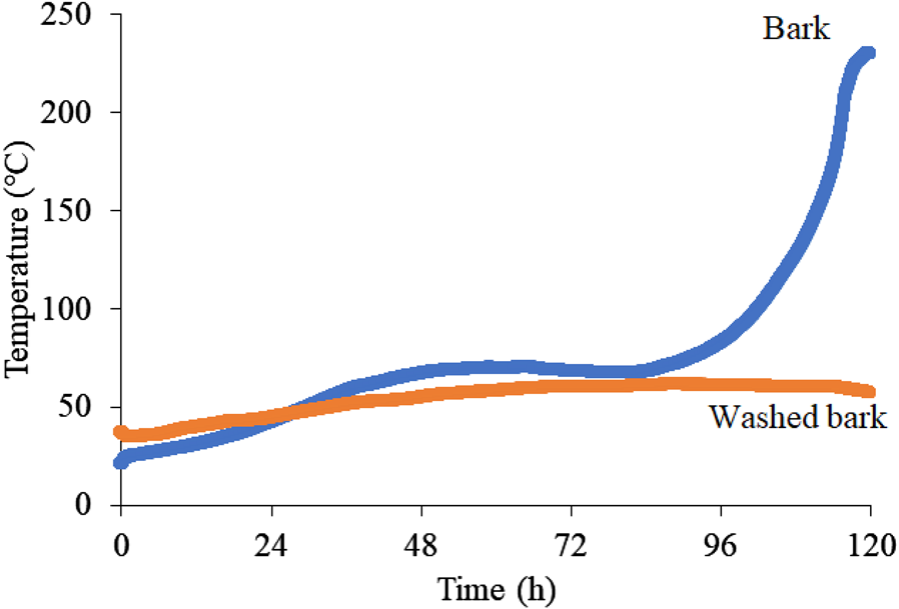
Self-heating of raw and washed bark chips

Sawdust and bark chips were compared to explore the raw material that caused self-heating. Figure 6 shows the self-heating experimental results for the sawdust and bark chips. The temperature of the sawdust increased to a maximum of 61 °C and decreased gradually from the maximum. In contrast, bark chips temperature showed more complicated behavior: it decreased slightly after rising to 70 °C, but then rose again, and finally reached over 200 °C. The higher reactivity of bark chips might be due to the inorganic matter contained in the material. Previous studies have reported that potassium could reduce the degradation temperature and encourage volatile matter release during torrefaction (Saleh et al. 2013; Saddawi et al. 2012). Therefore, by removing the ash from bark chips and comparing the thermal behavior with the raw material, it is thought that the effect of inorganic matter on self-heating may be investigated. Table 1 shows the ash contents of the raw and washed bark chips. The ash content contained in the washed bark chips was found to be ~ 0.4%d.b. lower than that contained in raw bark chips. The results of the inorganic analysis by inductively coupled plasma optical emission spectrometry are shown in Fig. 7. Washing bark chips reduced potassium by 0.1%d.b. and calcium by 0.05%d.b. Previous research also reported that large amounts of potassium could be removed by washing with water (Baxter et al. 1998) as most of the potassium in the biomass exists as water-soluble compounds or ions. The self-heating performance of the washed bark chips and raw bark chips is shown in Fig. 8. After the temperature of the raw materials was increased to 70 °C, the temperature gradually decreased before increasing again and finally reached 233 °C. For the water-washed bark chips, the temperature continued to increase gradually to 61 °C and then decreased. The washed bark chip temperature may have failed to increase due to the decrease in inorganic substances, especially potassium. Sujanti et al. (1999) investigated the effect of inorganic matter on spontaneous combustion of a coal and reported that the water washing and acid washing of inorganic substances raised the critical ambient temperatures of coal. Safar et al. (2019) also reported that the addition of K_2_CO_3_ (0.004 M or more) into the biomass intensified its oxidative reactivity due to the reduction of cellulose crystallinity. Thus, the small difference in amounts of inorganic substances was suggested to affect self-heating in this study.

**Table 1:** Characteristics of each sample

Sample	Higher heating value (MJ/kg)	Ash content (%d.b.)
Sawdust	19.83 ± 0.33	0.35 ± 0.01
Bark chips	19.05 ± 0.10	2.46 ± 0.18
Washed bark chips	–	2.09 ± 0.10
Torrefied bark chips	21.95 ± 0.13	4.23 ± 0.38

### Effect of moisture content on self-heating of bark chips

**Fig. 9:**
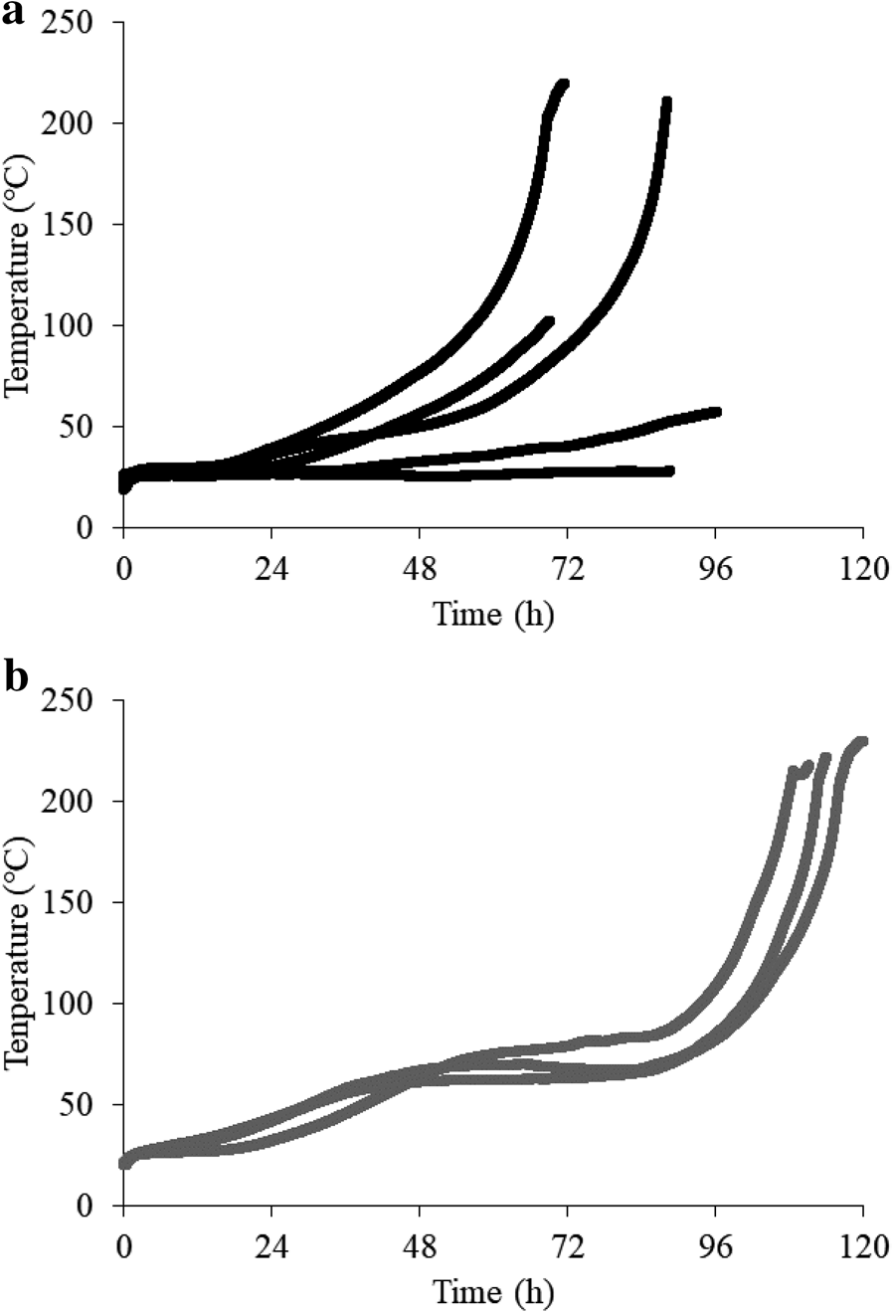
Self-heating with different moisture contents: **a** 10%w.b., **b** 40%w.b.

Self-heating experiments were conducted using bark chips with moisture contents of 10% and 40%w.b. to investigate the reactive repeatability under different moisture contents. Figure 9a, b shows the experimental results of self-heating at moisture content of 10 and 40%w.b., respectively. The temperature rise rate was unstable at a 10%w.b. moisture content as shown in Fig. 9a. Cases occurred for which the temperature rose to 200 °C sharply at 72 h or earlier, and other cases for which the temperature increase was negligible. At the moisture content of 40%w.b., as indicated in Fig. 9b, the temperature increase took longer than that at 10%w.b. moisture content. However, the heat generation behavior showed almost the same tendency in all experiments. The temperature continued to rise slowly until ~ 48 h, which is considered composting. Then, the temperature rose sharply at ~ 96 h and reached 200 °C at ~ 100 h. At a moisture content of 10%w.b., the effect of microbial heat generation on self-heating is small, and the main factor that causes self-heating is chemical oxidation. The self-heating of the chemical oxidation alone is unstable in this study because the heating generation by chemical oxidation is small at low temperatures. Meanwhile, the microbial heat generation was accelerated under a moisture content of 40%w.b., which enabled composting, and the temperature increase behavior had a reliable reproducibility. As a result, the torrefied bark chips had 21.95 MJ/kg of higher heating value and the quality was improved as shown in Table 1. Therefore, biochar production was demonstrated using the self-heating of wood biomass under atmospheric conditions.

## Conclusions

Self-heating experiments were performed using cedar sawdust and bark chips to produce biochar without the need for an external heat source. Consequently, the bark chips were found to be more active for self-heating than sawdust. Inorganic matter, especially potassium, was effective for self-heating because water-washed bark chips were not self-heated, and potassium was highly reduced. Furthermore, the self-heating experiments were carried out using bark chips with moisture contents of 10% and 40%w.b., respectively, to ensure stable reproducibility because the reactivity relies on moisture content. As a result, it was confirmed that the bark chips with a moisture content of 40%w.b. enabled reliable reproducibility during self-heating, and the produced biochar had an improved higher heating value of 21.95 MJ/kg. Therefore, this system can produce biochar by successive reactions, including biological oxidation, chemical oxidation, and ignition.

## Data Availability

All data analyzed during this study are included in this article.
